# Treatment of Different Forms of Headache

**Published:** 1875-10

**Authors:** Wm. Henry Day


Treatment of Different Forms of Headache.—*By Wm. Henry Day, M. D.*—In an interesting communication "On the Treatment of Headache from Intracranial Disease," Dr. Moxon refers to the scarcity of writers on headache, and says "it is not easy to get good guidance on the subject," an opinion in which I concur. Headache is such a common attendant on many diseases, especially the acute diseases, that it is naturally looked upon as a mere symptom.

I do not altogether agree with the classification adopted by the late Dr. Wright and some other writers. Thus, sick headache is classed as dependent on the digestive system, when it really belongs to the nervous system. The brain is the first organ to take offence. Clinical experience inclines the scale of probabilities in favor of a nervous and not a gastric origin. It is the nervous system which is primarily at fault, and the digestive system which is thrown out of order in consequence. Sick headache is a form of headache not always provoked by error or excess in diet, but belongs to nervous and excitable temperaments. It is commonly met with in children and delicate women, when the most regular diet has been observed, and the stomach and bowels are in no way disordered. I have known people, especially young persons of both sexes, suffer periodically from this form of headache, and attended even with bilious vomiting. They have clean tongues, clear eyes, pale urine, and healthy motions. Over brain-work, fatigue, late hours, and excitement will bring it on, and when these habits have been discontinued they will get relief, and go on for months without an attack. Some young women suffer severely before each catamenial period, and especially if they are subject to dysmenorrhœa. The vomiting of a little bile no more admits of any headache being classed as bilious than the vomiting of bile in severe sea sickness deserves to be ascribed to excess of bile in the system. In the case of a young gentleman recently under my care, of very nervous and anxious temperament, and who suffered from this sick-headache, I advised that between the intervals of the headache he should take bromide of ammonium in fifteen-grain doses, with five grains of citrate of iron in an ounce of water, twice a day. He continued this remedy for six weeks, and at the end of five months I heard that he had not suffered from any return. The excitement of traveling to London after this long interval of perfect health so upset his nervous system, that it brought on one of his worst attacks, attended with giddiness, confusion of ideas, and sickness. Headache, known as nervous headache, dependent on anæmia, neuralgia, leucorrhœa, mental and bodily fatigue, and prolonged anxiety, belongs to this class. It particularly attacks persons of delicate sensibility and weak organization. The pain may extend from the forehead to the occiput or the vertex, or one temple may be the chief seat of suffering.

Again, under the heading of bilious headache, Dr. Wright subdivided it into two forms, one arising from the "accumulation of bile in the system," and the other from "exuberant secretion of bile," which to my mind is an unnecessary refinement.

"Wisdom may entangle itself in over-niceness."

Among children I have classified headaches as follows :

1. Cerebral headache, attributable to injury or to chronic or acute inflammation.

2. Gastric headache, from intestinal and hepatic derangement, known as bilious headache.

3. Epileptic headache.

4. Febrile headache.

5. Headache from anæmia, neuralgia, etc., constituting nervous headache.

6. Headache depending on some intricate change in the cerebral membranes or tissues of the brain.

I have alluded to a type of the headache which I have termed "cerebral headache." It follows remotely on a slight blow or injury received at some earlier period which has escaped notice at the time, and this has led to a disturbance in the brain circulation and an interruption to its functions. The proposition I would endeavor to establish amounts to this—that an injury, barely sufficient in some instances to shake the contents of the brain, will in nervous or delicate children, or where hereditary predisposition to nervous disease is strong, awaken this character of headache, even if it does not lay the foundation of organic change. The head has in some instances only revealed its weakness when the strain of school-work has first been put upon it.

I have thought the headache of epilepsy and fever worth especial notice. The frequent occurence of epileptic seizures shakes and disturbes the whole cerebral mass, and by impairing the nervous structure causes headache having no very special features. I have met with some distressing cases of headache from this cause, lasting for days and even weeks together. Again, headache is a common accompaniment of fever in young and robust subjects, and cold affusion, and even the application of leeches, have been required to subdue it. In typhus and relapsing fever it is oftentimes severe, and is among the chief symptoms we have to combat. This is a form of headache symptomatic of a toxæmic condition of the blood, and from this cause we may have headache due to gout and rheumatism, syphilis, or Bright's disease.

The last form of headache, No. 6, is one possessing much interest. But to this wider field of inquiry, which I have already attempted elsewhere, the space at my disposal does not admit of any more than a passing allusion. I may observe that the first manifestations of illness are those of lethargy and inactivity. Headache is the first striking symptom that attracts notice, though the health may have been gradually declining, and the child losing flesh and strength. Bad food and clothing, foul air, and all the attributes of poverty, are among the common causes. Consumption, insanity, nervous disease, and syphilis, are often traceable on the father's or mother's side. Boys who show an aptitude for learning are sometimes victims to this form of headache. After any mental effort they become listless and variable in temper and manner, sometimes dull, and at other times fidgety and quarrelsome. The pulse is weak ; sighing and yawning are frequent symptoms ; the headache is constant, and the appetite poor. It is never so overwhelming in its severity as a sick headache, but is more continuous, and may last for days and even months together. The child easily becomes wearied, and falls off into sleep in the daytime, from which it does not awake refreshed. These cases are insidious in their origin, and, until headache is complained of, no decided symptoms attract notice. There is an obscure stage of debility and nervous prostration preceding this, and the evil is increased by continuance at school, deficient food and clothing, or severe moral discipline, on the ignorant assumption that the child is wayward and ill-tempered.

Bromide of potassium is a great remedy in these cases, and under its use the headache diminishes, the irritability passes off, and refreshing sleep is obtained. All the symptoms return with renewed force if the remedy is discontinued too soon. The special therapeutic effects of the drug are only to be gained by small doses, and iodide of potassium should be combined in still smaller doses with it. The bromide and iodide is the best combination where nervous exhaustion and headache are the leading symptoms. If persevered with, the headache and excitement abate, and so a safe pathway is made for cod-liver oil and other tonic remedies. We must subdue the pain before we can build up the general health. These peculiar brain derangements are successfully combated by the bromide of potassium, and the tendency to degenerative disease of the nerve-centres is arrested.

But this communication is chiefly invited by Dr. Moxon's testimony in favor of the hypodermic injection of morphia. In properly selected cases, this is one of the best, if it is not the only reliable remedy we possess. When the sickness and prostration are extreme, and nothing can be retained on the stomach, not so much as a little iced water—when the extremities are cold and the pulse feeble—when there is intolerance of light and sound, and the patient has been days without getting any relief, —the one-sixth of a grain of acetate of morphia for an adult will be sufficient to ensure sleep, and the patient will wake up without headache, if not well. Sometimes the addition of the 120th to the 80th of a grain of atropia will exert an antagonistic effect, and combat the tendency to sickness which morphia alone frequently excites.

I am glad to find that Dr. Moxon has succeeded with the hypodermic injection of morphia in some cases of headache from organic brain disease. Avowedly difficult as headache is to manage when it depends on structural change, pathological research has proved that hydatid tumors and morbid growths, involving the encephalon exist without causing pain. Even when it is most acute and continuous there are generally intermissions which afford the poor sufferer some temporary relief from his anguish.

There is another form of nervous headache happening to some women whose general health has been lowered by frequent childbearing, excessive catamenial discharges, or poverty and anxiety. In these people the sympathetic system is easily excited to disturbance through original delicacy of constitution in some instances, or if the functions of digestion and assimilation are overtaxed, the impression is conveyed to the sensorium by reflected irritation, and agonising headache is the result. The top of the head is hot, the pupils contracted, and the pulse weak and usually slow. There is a sensation as though something was scratching or alive within the head. Days before the attack comes on the patient is irritable and fidgety, and cannot settle down to her ordinary pursuits and occupation. Everything puts her out ; her reasoning is gone ; she is frequently sick, and her stomach loathes the sight of food. Life is such a burden that she can scarcely trust herself to be alone. This form of headache is due to meningeal irritation, and does not affect the cerebral mass itself unless the pain and suffering do not yield to treatment, and then the whole cerebral tissue becomes involved, and irritability is exchanged for indifference and despair.

Bromide of ammonium given early in the morning, before rising, with sal volatile, or small doses of iodide of potassium with the same aromatic stimulant, will have a good effect, whilst the general health requires attention in the intervals. Scruple doses of bromide of potassium in camphor mixture three times a day will give relief sometimes when quinine and bark in every conceivable form have failed. When it can be procured, change of air and scene do much for these sufferers, by diverting the mind and introducing fresh interests and occupation.

Some examples of this distressing headache have come before me, and in two cases, where the attacks were of frequent occurrence during a period of thirty years, they nearly ceased after the age of sixty. No explanation could be offered for their sudden cessation. And here I would just remark that when all remedies appear to fail in elderly people we should dissuade them from the use of stimulants. As they grow older they grow more indulgent, and increase their daily dose of wine, while they take less exercise, live in hot rooms, and are continually turning round their own narrow circle. They cannot take themselves from themselves. There are headaches even in old people which are purely functional, and yet so rebellious to treatment that we are often disposed to regard them as due to organic change.—*London Lancet.*

